# Supplementary material for: Allosteric regulation and crystallographic fragment screening of SARS-CoV-2 NSP15 endoribonuclease
Source: Nucleic Acids Res. 2023 Apr 28;51(10):5255–70. doi: 10.1093/nar/gkad314 (PMC10250223; doi:10.1093/nar/gkad314)
Supplement: gkad314_Supplemental_Files [file gkad314_supplemental_files.zip › Supplementary datasets.zip!/134/Refine_9/01-BUSTER/Cycle-2/shell.01/R_Rfree.html]

# Rxpct factors in resolution bins at cycle 0


|  |  |  |  |  |  |  |  |  |
| --- | --- | --- | --- | --- | --- | --- | --- | --- |
|  | **<d> (Å)**  Rxpct\_Work | Rxpct\_Free | Rxpct\_All | |  |  |  |  |
|  | 15.4464 | 0.3498 | 0.4403 | 0.3532 |  |
|  | 8.9180 | 0.2328 | 0.2541 | 0.2341 |  |
|  | 6.9078 | 0.2255 | 0.2524 | 0.2271 |  |
|  | 5.8382 | 0.2293 | 0.2954 | 0.2315 |  |
|  | 5.1488 | 0.1851 | 0.2028 | 0.1860 |  |
|  | 4.6573 | 0.1660 | 0.1994 | 0.1679 |  |
|  | 4.2841 | 0.1668 | 0.1901 | 0.1681 |  |
|  | 3.9882 | 0.1692 | 0.2240 | 0.1718 |  |
|  | 3.7463 | 0.1787 | 0.2237 | 0.1808 |  |
|  | 3.5436 | 0.1735 | 0.2066 | 0.1753 |  |
|  | 3.3707 | 0.1791 | 0.2182 | 0.1813 |  |
|  | 3.2208 | 0.1856 | 0.2117 | 0.1870 |  |
|  | 3.0893 | 0.1951 | 0.2098 | 0.1958 |  |
|  | 2.9727 | 0.1937 | 0.2074 | 0.1942 |  |
|  | 2.8683 | 0.1961 | 0.2009 | 0.1963 |  |
|  | 2.7743 | 0.1977 | 0.2316 | 0.1992 |  |
|  | 2.6889 | 0.1961 | 0.2048 | 0.1966 |  |
|  | 2.6109 | 0.2044 | 0.2212 | 0.2053 |  |
|  | 2.5394 | 0.2147 | 0.2366 | 0.2160 |  |
|  | 2.4734 | 0.2361 | 0.2630 | 0.2376 |  |
|  | **Overall** | 0.1974 | 0.2267 | 0.1989 |  |

# Rxpct factors in resolution bins at cycle 1


|  |  |  |  |  |  |  |  |  |
| --- | --- | --- | --- | --- | --- | --- | --- | --- |
|  | **<d> (Å)**  Rxpct\_Work | Rxpct\_Free | Rxpct\_All | |  |  |  |  |
|  | 15.4464 | 0.3500 | 0.4408 | 0.3535 |  |
|  | 8.9180 | 0.2329 | 0.2541 | 0.2342 |  |
|  | 6.9078 | 0.2258 | 0.2528 | 0.2274 |  |
|  | 5.8382 | 0.2292 | 0.2944 | 0.2314 |  |
|  | 5.1488 | 0.1851 | 0.2029 | 0.1861 |  |
|  | 4.6573 | 0.1661 | 0.1994 | 0.1680 |  |
|  | 4.2841 | 0.1665 | 0.1892 | 0.1677 |  |
|  | 3.9882 | 0.1689 | 0.2225 | 0.1713 |  |
|  | 3.7463 | 0.1781 | 0.2226 | 0.1802 |  |
|  | 3.5436 | 0.1731 | 0.2064 | 0.1749 |  |
|  | 3.3707 | 0.1787 | 0.2157 | 0.1807 |  |
|  | 3.2208 | 0.1852 | 0.2121 | 0.1867 |  |
|  | 3.0893 | 0.1948 | 0.2103 | 0.1955 |  |
|  | 2.9727 | 0.1934 | 0.2087 | 0.1940 |  |
|  | 2.8683 | 0.1957 | 0.1985 | 0.1958 |  |
|  | 2.7743 | 0.1972 | 0.2352 | 0.1990 |  |
|  | 2.6889 | 0.1962 | 0.2083 | 0.1969 |  |
|  | 2.6109 | 0.2045 | 0.2208 | 0.2054 |  |
|  | 2.5394 | 0.2143 | 0.2363 | 0.2157 |  |
|  | 2.4734 | 0.2355 | 0.2585 | 0.2368 |  |
|  | **Overall** | 0.1972 | 0.2265 | 0.1987 |  |

# Rxpct factors in resolution bins at cycle 2


|  |  |  |  |  |  |  |  |  |
| --- | --- | --- | --- | --- | --- | --- | --- | --- |
|  | **<d> (Å)**  Rxpct\_Work | Rxpct\_Free | Rxpct\_All | |  |  |  |  |
|  | 15.4464 | 0.3501 | 0.4408 | 0.3536 |  |
|  | 8.9180 | 0.2330 | 0.2537 | 0.2343 |  |
|  | 6.9078 | 0.2262 | 0.2536 | 0.2278 |  |
|  | 5.8382 | 0.2291 | 0.2936 | 0.2313 |  |
|  | 5.1488 | 0.1853 | 0.2033 | 0.1863 |  |
|  | 4.6573 | 0.1660 | 0.1994 | 0.1679 |  |
|  | 4.2841 | 0.1663 | 0.1896 | 0.1676 |  |
|  | 3.9882 | 0.1686 | 0.2228 | 0.1711 |  |
|  | 3.7463 | 0.1774 | 0.2234 | 0.1795 |  |
|  | 3.5436 | 0.1730 | 0.2069 | 0.1748 |  |
|  | 3.3707 | 0.1784 | 0.2161 | 0.1805 |  |
|  | 3.2208 | 0.1855 | 0.2128 | 0.1870 |  |
|  | 3.0893 | 0.1946 | 0.2114 | 0.1954 |  |
|  | 2.9727 | 0.1934 | 0.2090 | 0.1940 |  |
|  | 2.8683 | 0.1955 | 0.1992 | 0.1957 |  |
|  | 2.7743 | 0.1970 | 0.2353 | 0.1988 |  |
|  | 2.6889 | 0.1961 | 0.2079 | 0.1967 |  |
|  | 2.6109 | 0.2043 | 0.2205 | 0.2052 |  |
|  | 2.5394 | 0.2145 | 0.2359 | 0.2158 |  |
|  | 2.4734 | 0.2352 | 0.2601 | 0.2366 |  |
|  | **Overall** | 0.1971 | 0.2268 | 0.1986 |  |

# Rxpct factors in resolution bins at cycle 3


|  |  |  |  |  |  |  |  |  |
| --- | --- | --- | --- | --- | --- | --- | --- | --- |
|  | **<d> (Å)**  Rxpct\_Work | Rxpct\_Free | Rxpct\_All | |  |  |  |  |
|  | 15.4464 | 0.3428 | 0.4201 | 0.3458 |  |
|  | 8.9180 | 0.2244 | 0.2546 | 0.2262 |  |
|  | 6.9078 | 0.2171 | 0.2644 | 0.2198 |  |
|  | 5.8382 | 0.2181 | 0.3107 | 0.2212 |  |
|  | 5.1488 | 0.1777 | 0.2067 | 0.1792 |  |
|  | 4.6573 | 0.1612 | 0.1975 | 0.1632 |  |
|  | 4.2841 | 0.1645 | 0.1909 | 0.1659 |  |
|  | 3.9882 | 0.1616 | 0.2190 | 0.1643 |  |
|  | 3.7463 | 0.1683 | 0.2259 | 0.1710 |  |
|  | 3.5436 | 0.1703 | 0.2087 | 0.1723 |  |
|  | 3.3707 | 0.1755 | 0.2168 | 0.1777 |  |
|  | 3.2208 | 0.1852 | 0.2221 | 0.1872 |  |
|  | 3.0893 | 0.1953 | 0.2196 | 0.1964 |  |
|  | 2.9727 | 0.1948 | 0.2095 | 0.1954 |  |
|  | 2.8683 | 0.1984 | 0.2043 | 0.1987 |  |
|  | 2.7743 | 0.1976 | 0.2393 | 0.1995 |  |
|  | 2.6889 | 0.1967 | 0.2019 | 0.1969 |  |
|  | 2.6109 | 0.2033 | 0.2183 | 0.2042 |  |
|  | 2.5394 | 0.2153 | 0.2261 | 0.2159 |  |
|  | 2.4734 | 0.2337 | 0.2555 | 0.2350 |  |
|  | **Overall** | 0.1928 | 0.2283 | 0.1946 |  |
